# The devastating impact of conflict in Sudan on mental health services: a rapid review

**DOI:** 10.1007/s44192-025-00207-4

**Published:** 2025-06-02

**Authors:** Wagd Mutaz Osman, Leena Abdelghani Mohamed, Abdulqadir J. Nashwan

**Affiliations:** 1 Faculty of Medicine, Almadain College for Medical Sciences and Technology, Khartoum, Sudan; 2 Master of Public Health – International University of Africa, Khartoum, Sudan; 3 Hamad Medical Corporation, 3050, Doha, Qatar; 4 Department of Public Health, College of Health Sciences, QU Health, Qatar University, Doha, Qatar

**Keywords:** Sudan crisis, Mental health, Healthcare infrastructure, Political instability, Humanitarian assistance

## Abstract

Sudan's healthcare system teeters on the brink of collapse as the country continues to reel from political instability and conflict that escalated in April 2023. The latest outbreak of violence between the national army and the Rapid Support Forces has exacerbated an already fragile healthcare infrastructure. This review provides a narrative synthesis of the existing literature and reports on the current state of mental health services in Sudan. The ongoing conflict has triggered widespread displacement, economic instability, and trauma, which have in turn contributed to increased rates of mental illness. While mental health services were limited even before the conflict, the current crisis has significantly reduced access to care, especially for vulnerable populations. In this review, we highlight the urgent need for comprehensive mental health interventions and sustained support for healthcare providers under increasingly dangerous conditions.

## Background

Even prior to the 2023 conflict, the economic landscape of Sudan crumbled in 2018, and civilians swarmed the streets in an unparalleled outpouring of dissent [1, 2]. Their clamor resounded with demands for economic rejuvenation and the ousting of President al-Bashir. A hybrid military-civilian administration took form after these demonstrations [1]. However, this government's tenure was short-lived, as it was toppled in 2021 by a coup d'état led by General Abdel Fattah al-Burhan [3]. Since that pivotal moment, Sudan's governance has been commandeered by a council of high-ranking military officers, with two towering figures at the vortex of the maelstrom: General Abdel Fattah al-Burhan, who heads the Sudanese Armed Forces (SAF) and serves as the nation's interim president, and his second-in-command, General Mohamed Hamdan Dagalo, the chieftain of the Rapid Support Forces (RSF), a formidable paramilitary organization [4]. To steer the country back toward civilian governance, the military junta inked an agreement stipulating a transition in April 2023. However, simmering disagreements between the two generals concerning the mechanics of the transition fanned the flames of tension, culminating in a violent clash between their respective military factions on April 15 [5]. This tussle for supremacy emanates from the thirst to wield control over a nation perched at a geopolitical crossroads, bridging North Africa, the Sahel, the Horn of Africa, and the Red Sea. Furthermore, Sudan's abundant natural resources make it a coveted prize in this power struggle [6]. Historically, the roots of Sudan’s lamentable trajectory are deeply entrenched. Despite their political inclinations, successive central administrations have been embroiled in skirmishes on the outskirts while safeguarding the central riverine heartland from the scourge of violence. The RSF represents the latest incarnation in a historical tapestry woven with militias deployed as government instruments to quell regional dissent [7]. 

### Humanitarian consequences

The situation in Sudan has deteriorated significantly since the onset of the conflict on April 15, 2023. As of March 2025, the conflict has claimed the lives of at least 25,000 people and injured over 42,000, according to updated data from the Protection Cluster [8]. The humanitarian crisis has escalated, with over 11.5 million people displaced, seeking safety within and beyond Sudan's borders. The conflict's toll on civilians has been devastating, with reports of deaths and widespread sexual violence. Moreover, essential services have been severely disrupted, including access to clean water, following the takeover of four water stations by warring parties [8].

Furthermore, access to medical care has become alarmingly scarce. Since the conflict began, at least 67% of hospitals in conflict zones have ceased operations, according to the Sudan Doctors’ Trade Union [7]. Of the 89 major hospitals across Sudan's capital and states, 60 are no longer functional, while the remaining 29 are operating under partial capacity, often limited to emergency services. These facilities risk imminent closure due to a severe shortage of medical staff, supplies, water, and electricity [4]. At least 17 hospitals have been shelled, and 21 have been forcibly evacuated since the war began. Moreover, 11 ambulances have been attacked, and others have been denied passage for patient transport [7].

The disruption of essential supplies has also had a dire economic impact. The looting and bombing of pharmaceutical and food factories, as well as significant import companies in Khartoum, have caused a complete halt in the supply of daily necessities. This has dramatically increased food prices, contributing to high inflation rates and placing millions at risk of starvation [9].

### Overview of mental health services in Sudan

The World Health Organization (WHO) defines mental health as “a state of well-being in which an individual realizes his or her abilities, can cope with the normal stresses of life, can work productively, and can contribute to his or her community” [10]. In this positive sense, mental health is the foundation for individual well-being and the effective functioning of a community [11].

However, mental health is not a priority in many developing countries in terms of the presence of policies, services, and research. The nation's financial difficulties and political instability have made providing health services, especially mental health treatment, difficult. The civil wars in Sudan have been associated with a rise in mental illnesses such as PTSD (post-traumatic stress disorder) and depression, particularly in young people and women [12]. The government, on the other hand, changed its funding priorities in response to the high rate of violence and gave mental health even less attention.

The Sudanese government has not focused heavily on creating mental health legislation over the past 20 years. The government's previous mental health law was passed in 1998, so it must be updated. Since then, the strategy has undergone slight reformulation in 2006–2008, but it is insufficient. Although there is currently a national mental health authority operating at the federal level under the auspices of preventive medicine, mental health treatments are neither organized nor accessible at the primary level of care [11]. 

### Mental health services in Sudan: infrastructure and facilities

Sudan has three different kinds of mental healthcare facilities: mental health centers, forensic inpatient facilities, and local psychiatric units. Each has a distinct capacity for treating patients with specialized staff and tools**.** In Sudan, there are only two mental health facilities, both serving Khartoum's central population. This causes a huge number of patients to make trips of significant distances to get psychological care. These medical clinics give ongoing consideration a joined all out of 0.86 beds per 100,000 populaces, or 327 (326.8) beds [11].

From 2007 to 2010, these hospitals averaged 24,322 patients per year. Taha Bashar, one of the few hospitals, has no phones or ambulances to help patients [13]. All of Sudan’s 200 forensic inpatient beds are situated within mental health facilities that are part of a prison system [11].

According to the most recent data, approximately 28,880 patients are treated annually. Police often forcefully admit the majority of patients after violent outbursts. At least 760 inpatient beds are provided by community-based psychiatric units, making them the most prevalent form of mental health care. Twenty community-based psychiatric units occupy a critical juncture in the mental healthcare landscape. However, these units, which serve as the vanguards of mental health treatment and are the most accessible options for the majority of the citizenry, find themselves sorely under-equipped and staffed by the least seasoned professionals [13].

### Damage to infrastructure

The Sudanese health system is on the verge of collapse as instability in the country enters its second month despite numerous broken peace deals. Seventy percent of the hospitals in the crisis zones are inoperable because hospitals have been targeted. The delivery of drugs and medical equipment to conflict-affected areas to treat people has also been hampered by border closures. With no end in sight, the battle has rendered the circumstances for civilians and medical personnel intolerable. Most humanitarian relief operations have been suspended, and most hospitals in the impacted areas only provide emergency care to injured people.

The relentless conflict has devastated the nation’s infrastructure. Aerial bombardments have turned healthcare facilities into rubble and destroyed vital water pipelines. With no alternatives, countless individuals have turned to the Nile River for bathing and drinking, exposing themselves to numerous life-threatening parasitic infections and waterborne diseases. The healthcare crisis is severe, and this is just the tip of the iceberg. The psychological effects on the population will unfold in the coming months [14]. A disturbing account from the manager of Al-Tigany Al-Mahy Mental Health Hospital, a key mental health facility in Khartoum, sheds light on the gravity of the situation:"Since the onset of this war, the Rapid Support Forces have established a menacing presence in Al-Tijani Al-Mahi Hospital. They seized control of the hospital under the threat of violence following the evacuation of patients for their safety. On May 19, they embarked on a rampage within the hospital, pilfering money, decimating laboratory equipment, and looting offices, warehouses, and the pharmacy. They made off with computers, air conditioners, generators, and more. They even purloined a transport vehicle. The hospital was subsequently abandoned and left vulnerable to further looting. After hospital staff regrouped to re-establish operations in a grim turn of events, the Rapid Support Forces again overran the hospital with an even larger contingent on the evening of May 19″ [15].

The Federal Ministry of Health has sounded the alarm, highlighting an urgent and overarching need for medical supplies. The clarion call emphasizes the need for medications to treat patients battling tumors, cancers, and other life-threatening ailments, emergency pharmaceuticals, and analgesics. A pressing need also exists for cardiac medications, laboratory equipment, and additional medicines. To optimize the supply chain under these difficult circumstances, state warehouses have been strategically positioned in the Red Sea, the Nile River, and the Gezira [16].

### Impact on mental health professionals

Sudan has approximately 34 psychiatrists, 425 psychologists, and 366 social workers. Very few psychiatrists operate in Sudan’s rural areas. As of 2020, 878 professionals across public, private, and NGO sectors manage mental health in Sudan [17]. Many young doctors move abroad to further their knowledge, experience, and salaries. This causes a shortage of health professionals, often stretched beyond their capacity. Illustrating this, data shows that Sudan has only 2.05 total mental health professionals per 100,000 people in a country with a population of nearly 45 million people [17].

The military presence inside medical institutions or their vicinity, or using them as launching pads for missiles, turning them into a battlefield, has endangered medical personnel and patients, led to a complete cessation of service, and led to the evacuation and shutdown of these institutions [18].

Since the conflict began, 38 Medical field personnel have been killed [19]. More were injured or detained, or displaced. This loss of personnel poses one of the main obstacles to receiving proper health care. Healthcare personnel who were able to reach operating hospitals and emergency rooms faced the stress and workload of working in such unsafe, unprepared hospitals and with the constant fear of being killed, injured, or detained, hindering the already weak response and attention to mental health problems [18].

Under these circumstances, the mental toll on medical personnel is enormous. An initiative, ‘Hidak’—Sudanese slang for ‘by your side’—led by a group of psychiatry specialists aims to provide online support group sessions and consultations targeting medical workers. Even these might not be accessible due to the unstable network [20]. More attention needs to be directed towards the burden and stresses of current events on the mental health of healthcare personnel.

### Escalation of mental health issues among the population

The disruption of civil unrest that has swept across the nation has dealt a devastating blow to healthcare services, with mental healthcare bearing the brunt of this onslaught. Considering the heightened prevalence of mental disorders among those who have been trapped in the crossfire of conflict and violence, the absence of mental healthcare services in the embattled regions is especially alarming. Moreover, the supply chain for indispensable resources, most notably food and medication, has been severely compromised. The citizens of Sudan are gripped by trepidation as they face a harrowing humanitarian crisis that looms ominously on the horizon, threatening to escalate further.

In Khartoum State, 13% of secondary school students are affected by depression and anxiety. Pregnant women are not immune to this mental health crisis; a staggering 23% have been diagnosed with prenatal mental disorders across primary care facilities and communities within Sudan’s capital, Khartoum. The internally displaced population exhibits an even more distressing picture, with an overwhelming 53% suffering from mental disorders, including major depressive disorder (24.3%), generalized anxiety disorder (23.6%), social phobia (14.2%), and PTSD (14.2%). The prevalence of severe mental disorders among internally displaced individuals is pegged at 1.5%. However, there is a glaring lack of data about attempted or completed suicides, as well as drug and alcohol abuse [21].

The ongoing conflict has erected a formidable obstacle for relief organizations attempting to deliver their aid. Women and children, being the most vulnerable segments of society, are engulfed in profound suffering. The threats of hunger and illness loom over the region, with vaccination initiatives facing significant disruptions. Only a handful of school-going children can pursue an education, and the threat of abduction and abuse at the hands of soldiers looms large. Relief aid in the camps is sporadic at best, and displaced women, lacking assets or skills, are reduced to scraping by through domestic labor, begging, petty trading, or resorting to beer-brewing and prostitution (the latter two being illicit activities). Children are left to their own devices or forced into labor, where they are exploited. Mental afflictions are the inevitable fallout of this desperate situation. Displaced children exhibit aggressive tendencies, while their counterparts in conflict zones are tormented by trauma and anxiety [22].

### Accessibility and utilization of mental health services

As per an incisive report compiled by the Sudanese Psychiatrists Association, mental illness continues to be shrouded in a pall of societal stigma [23]. This cloud of misunderstanding and discrimination casts a long shadow, often resulting in a reluctance among those afflicted to seek help until the illness reaches advanced stages. Furthermore, the coverage of mental health services is woefully inadequate, rendering access to necessary care a formidable challenge for many [23]. Adding fuel to the fire, the nation has been beleaguered by protracted conflicts and political upheavals, spawning large-scale population displacements. These dislocations have engendered many immediate and long-term health perils, which weigh most heavily on those seeking out an existence in makeshift settlements. These communities, which are often hastily cobbled together, lack the infrastructure and amenities necessary to safeguard the health and well-being of their inhabitants. In such a tenuous environment, the cumulative stress and uncertainty breed a fertile ground for mental health disorders. This scenario calls for an urgent and multifaceted response, encompassing both the expansion of mental health services and a concerted effort to dismantle the stigma that enshrouds mental illness.

### Challenges in accessing mental health services during conflict

Before the eruption of the current conflict, a pernicious geographic disparity already plagued the accessibility of mental health care in Sudan. Of the nation's 18 states, a mere 12 boast what can be termed “fully-equipped psychiatric hospitals” staffed with proficient psychiatric personnel, according to a study published in 2020 by Osman and colleagues [23]. The distribution of these hospitals is highly uneven; half of them are concentrated in Khartoum, while the remainder, dispersed among six other states, operate under the stewardship of non-specialist medical practitioners, clinical psychologists, or medical assistants. Among the major mental health facilities in Khartoum, the Al-Tigany Al-Mahy Mental Health Hospital has borne a particularly grievous blow in the wake of the conflict [15]. Once a sanctuary for those seeking mental health care, the hospital has been evacuated and is now occupied by the Rapid Support Forces, repurposed as a military outpost. The Mental Health Atlas of 2020 paints a bleak picture of the financial burden that accompanies mental health care in Sudan [17]. Those grappling with mental disorders bear the brunt of costs for services and medications, which are typically paid"mostly or entirely out of pocket."The escalation of conflict has wrought further devastation on infrastructure and livelihoods, eroding the already tenuous ability of individuals to shoulder the financial burden of their treatments.

### Changes in mental health services’ utilization patterns

The beliefs and stigma around mental illness, resorting to alternative treatments such as religious and traditional healers, centralization of mental health services, inadequate number of mental health staff, cost of medications, and mental health not being a priority by policymakers were the main barriers to the utilization of mental health services [24]. In the first days of the conflict, the Federal Ministry of Health issued a list of all specialists and consultants willing to provide support and counseling services online; this appears to be the most helpful and applicable form of treatment in such circumstances. Many patients continue their treatments without further consultations due to the inability to reach hospitals and clinics or connection problems suspending contact with their doctors. Many more stopped taking their medications altogether, whether because of the unavailability of these drugs due to the overall shortage of medicines or because of the economic weight of the war and prioritizing other necessities like food and housing.

Recent assessments have highlighted the rapid deterioration of Sudan’s mental health services amid ongoing conflict. A multi-sectoral study by Abdelrahim et al. identified widespread psychological distress driven by violence and displacement alongside major barriers such as stigma, lack of awareness, and an acute shortage of trained professionals. Similarly, Elhadi et al. emphasized how conflict has crippled healthcare infrastructure, particularly mental health facilities, and called for urgent, coordinated interventions to address trauma and service gaps [25].

## Conclusion

Over the past two decades, mental health has languished on the periphery of the Sudanese government’s agenda, with laws and services in this critical domain garnering scant attention. The relentless storm of armed conflict that now besieges Sudan has further laid waste to an already beleaguered mental health landscape as the nation mourns the loss of its principal mental health facilities. Mental health professionals, who are the very bulwark against this mental health crisis, find themselves navigating treacherous waters. They are bereft of the assurance of a safe working milieu and burdened by the astronomical stress and a demanding workload. Moreover, many among their ranks are themselves victims of displacement. The situation is further exacerbated by a pervasive scarcity of medicines, a lifeline for those contending with mental health disorders. Considering this dire scenario, mental health must be catapulted to the forefront of the nation's priorities. An urgent clarion call must be issued for sweeping measures to alleviate the burdens and stresses that the tumultuous current events have exacted on the mental well-being of the populace at large, as well as on the valiant healthcare personnel who labor relentlessly under the grimmest and forbidding of circumstances. The fabric of the nation’s mental health, threadbare and frayed, calls out for mending through concerted and compassionate efforts.

## Data Availability

Not applicable.
